# Transvenous Lead Extraction in Patients with Cardiac Implantable Device: The Impact of Systemic and Local Infection on Clinical Outcomes—An ESC-EHRA ELECTRa (European Lead Extraction Controlled) Registry Substudy

**DOI:** 10.3390/biology11040615

**Published:** 2022-04-18

**Authors:** Igor Diemberger, Luca Segreti, Christopher A. Rinaldi, Jesper Hastrup Svendsen, Andrzej Kutarski, Arwa Younis, Cécile Laroche, Christophe Leclercq, Barbara Małecka, Przemyslaw Mitkowski, Maria Grazia Bongiorni, On behalf of the ELECTRa Investigators

**Affiliations:** 1 Institute of Cardiology, Department of Experimental, Diagnostic and Specialty Medicine, University of Bologna, 40138 Bologna, Italy; 2 Cardiology Unit, IRCCS, Azienda Ospedaliero-Universitaria Di Bologna, 40138 Bologna, Italy; 3 Cardiology Department, University Hospital of Pisa, Via Paradisa 2, 56124 Pisa, Italy; lucasegreti@gmail.com (L.S.); m.g.bongiorni@med.unipi.it (M.G.B.); 4 Cardiology Department, 6th Floor East Wing, Guy’s & St Thomas’ Hospitals, Westminster Bridge Rd, London SE1 7EH, UK; c.rinaldi@virgin.net; 5 Department of Cardiology, The Heart Centre, Rigshospitalet, Copenhagen University Hospital, 1165 Copenhagen, Denmark; jesper.hastrup.svendsen@regionh.dk; 6 Department of Clinical Medicine, Faculty of Health and Medical Sciences, University of Copenhagen, 1165 Copenhagen, Denmark; 7 Department of Cardiology, Medical University of Lublin, ul. Jaczewskiego 8, 20-954 Lublin, Poland; a_kutarski@yahoo.com; 8 Heart Center/Sheba Medical Center Tel Hashome, Sackler School of Medicine Tel Aviv University, Tel Aviv 69978, Israel; or.younis@gmail.com; 9 EURObservational Research Programme (EORP), Scientific Division, European Society of Cardiology (ESC), 2035 Route des Colles, Sophia Antipolis, 06903 Valbonne, France; claroche@escardio.org; 10 Service de Cardiologie et Maladies Vasculaires CHU Pontchaillou, 2, Rue Henri le Guilloux, CEDEX 09, 35033 Rennes, France; christophe.leclercq@chu-rennes.fr; 11 Department of Electrocardiology, The John Paul II Hospital Institute of Cardiology, Jagiellonian University Medical College, 80 Prądnicka St., 31-202 Kraków, Poland; barbara_malecka@go2.pl; 12 1st Department of Cardiology, Karol Marcinkowski University of Medical Sciences Head Electrotherapy Laboratory, University Hospital ul. Dluga 1/2, 61-848 Poznan, Poland; przemyslaw.mitkowski@ump.edu.pl

**Keywords:** CIED, survival, endocarditis, infection, pacemaker, defibrillator

## Abstract

**Simple Summary:**

According to our analysis of the multicenter EORP ELECTRa (European Lead Extraction ConTRolled) Registry, about one-third of the candidates to complete removal of an implantable pacemaker/defibrillator because of device infection, through standard transvenous lead extraction, presents a systemic infection. Systemic infection is associated with a higher incidence of major in-hospital complications overall and a strong trend for procedure-related complications. Moreover, systemic infection is associated with increased procedure-related and non-procedure-related in-hospital mortality. Patients with cardiac implantable devices (CIED)-related infection with systemic vs. local involvement present different characteristics suggesting that in a relevant subgroup of patients the infection can be systemic from the beginning, without progression from CIED pocket.

**Abstract:**

Background: Infections of cardiac implantable devices (CIEDI) have poor outcomes despite improvement in lead extraction (TLE) procedures. Methods: To explore the influence of CIEDI on the outcomes of TLE and the differences between patients with systemic (Sy) vs. local (Lo) CIEDI, we performed a sub-analysis of the EORP ELECTRa (European Lead Extraction ConTRolled) Registry. Results: Among 3555 patients enrolled by 73 centers in 19 Countries, the indication for TLE was CIEDI in 1850: 1170 with Lo-CIEDI and 680 with Sy-CIEDI. Patients with CIEDI had a worse in-hospital prognosis in terms of major complications (3.57% vs. 1.71%; *p* = 0.0007) and mortality (2.27% vs. 0.49%; *p* < 0.0001). Sy-CIEDI was an independent predictor of in-hospital death (H.R. 2.14; 95%CI 1.06–4.33. *p* = 0.0345). Patients with Sy-CIEDI more frequently had an initial CIED implant and a higher prevalence of comorbidities, while subjects with Lo-CIEDI had a higher prevalence of previous CIED procedures. Time from signs of CIEDI and TLE was longer for Lo-CIEDI despite a shorter pre-TLE antibiotic treatment. Conclusions: Patients with CIEDI have a worse in-hospital prognosis after TLE, especially for patients with Sy-CIEDI. These results raise the suspicion that in a relevant group of patients CIEDI can be systemic from the beginning without progression from Lo-CIEDI. Future research is needed to characterize this subgroup of patients.

## 1. Introduction

The number and complexity of cardiovascular implantable electrical devices (CIED) used in current clinical practice are progressively increasing due to the improvement in technology and expansion of indications [1]. This trend is accompanied by a rise in CIED-related complications, in particular CIED infections (CIEDI) which have a dramatic impact on patient prognosis and healthcare associated costs [2]. The results of the European Lead Extraction ConTRolled Registry (ELECTRa) [3], a large multicentre prospective registry of consecutive patients candidates for transvenous lead extraction (TLE), showed that despite a high success rate of TLE procedures with low procedure-related complications (mortality 0.5%, 95% CI 0.3–0.8%), in-hospital overall mortality was not negligible (1.4%, 95% CI 1.1–1.9%) with systemic CIEDI being the strongest independent risk factor (OR 4.93, 95% CI 2.72–8.93%; *p* < 0.0001) [3]. These findings underline the complex management of candidates to TLE, especially in the case of CIEDI [2,4]. The current sub-analysis was aimed at defining the impact of CIEDI on the outcomes of TLE and the differences between patients with local vs. systemic infection. We also tried to identify the predictors of in-hospital mortality in the subgroup of candidates to TLE for CIEDI, with the strong belief that these data can be helpful in planning the pre- and post-TLE management of patients with CIEDI and possibly influence CIEDI prophylaxis. 

## 2. Methods

### 2.1. The ELECTRa Registry 

The ELECTRa registry was a large multicentre prospective registry of consecutive patients undergoing TLE. The registry was conducted by the European Heart Rhythm Association (EHRA). The executive committee in cooperation with the EURObservational Research Program (EORP) provided the study design, protocol, and the scientific leadership of the registry under the responsibility of the EHRA Scientific Initiatives Committee (SIC). EHRA affiliated centres participating in the registry were required to recruit all consecutive patients with an indication for TLE (excluding those patients primarily requiring surgical extraction) in their institution. All subjects provided written informed consent prior to the extraction procedure. No specific protocol or recommendations regarding technique were made for the TLE procedure. A detailed description of the study design and of the electronic case report form (e-CRF) has been previously described [5]. The ELECTRa registry complies with the Declaration of Helsinki, a locally appointed ethics committee for each centre has approved the research protocol and informed consent has been obtained from all the subjects (or their legally authorized representative).

### 2.2. Sub-Study Characteristics: Patient Selection, Endpoints

From 1 November 2012 to 31 May 2014, 73 EHRA affiliated centres from 19 countries participated in the ELECTRa registry. A total of 3555 consecutive patients were enrolled, of whom 3510 (98.7%) underwent TLE. Among this cohort of patients 1850/3510 (52.7%) underwent TLE for CIEDI, either local (Lo-CIEDI) or systemic (Sy-CIEDI) [3]. Notably, discrimination between Lo-CIEDI and Sy-CIEDI is not a straightforward process since it is heavily influenced by the time passed from the first sign of CIEDI and referral for TLE, the type and timing of diagnostic tests performed (e.g., early/late culturing with/without previous antibiotics) and their interpretation (e.g., masses found at echocardiography can be either thrombus or vegetations). Notably, also current guidelines and consensus documents underline this issue acknowledging the limitations of modified Duke Criteria in this setting [6,7,8]. For these reasons the ELECTRa scientific board decided to adopt an operative definition of Lo-CIEDI vs. Sy-CIEDI based on investigator discretion, analyzing the complete clinical picture on the basis of the modified Duke Criteria [9,10]. 

The primary endpoint of the current analysis was to describe the characteristics associated with CIEDI in candidates to TLE and to evidence the differences between patients with CIEDI Lo-CIEDI vs. Sy-CIEDI in terms of baseline characteristics, indications, and outcomes for TLE and in-hospital mortality. The main secondary endpoint was the identification of predictors of mortality before discharge considering the presence of Lo-/Sy-CIEDI.

### 2.3. Statistical Analysis

Results were summarized by absence/presence and type of CIEDI (Lo-CIEDI vs. Sy-CIEDI). Continuous variables were reported as mean ± standard deviation (SD) or as median and inter-quartile range (IQR). Among-group comparisons were made using a non-parametric test (Mann-Whitney test). Categorical variables were reported as counts and percentages (without missing values if applicable). Among-group comparisons were made using a Chi-square test or the Fisher’s exact test (if any expected cell count was less than five). A stepwise multiple Cox regression was used to determine the predictors of in-hospital all-cause mortality and major complications including death selecting into the models all the respective candidate variables (variables with *p* < 0.05 in univariate, except those with more than 20% of missing data) and the type of CIEDI (Lo- Vs. Sy-) (the analysis of these predictors in the overall population was previously published [3]). A significance level of 0.05 was required to allow a variable into the model (SLENTRY = 0.05), and a significance level of 0.05 was required for a variable to remain in the model (SLSTAY = 0.05). No interaction was tested. A Hosmer and Lemeshow Goodness-of-Fit test was used to verify that the model was optimal. A two-sided *p*-value of 0.05 was considered statistically significant. All the analyses were performed by the Scientific Division of the EURObservational Research Programme (EORP) using SAS statistical software version 9.4 (SAS Institute, Inc., Cary, NC, USA).

## 3. Results

### 3.1. Patient Population and Baseline Clinical Characteristics

From 1 November 2012 to 31 May 2014, 73 European centres from 19 countries participated in the study. A total of 3555 consecutive patients were enrolled in the study: 1850/3555 (52.04%) underwent TLE for CIEDI and 1634/3555 (45.96%) for other non-infective indications, mainly to add/substitute a CIED lead. The remaining patients were not included in the analysis since 44 did not underwent TLE and in 26 attributions to a specific subgroup was not possible despite post-hoc CRF revision. Patients with CIEDI were older (age ≥ 65 years: 69.24% vs. 46.33%; *p* < 0.0001) and more frequently males (77.95% vs. 65.97%; *p* < 0.0001) with a higher prevalence of comorbidities such as hypertension (59.35% vs. 48.42%; *p* < 0.0001), diabetes (27.61% vs. 16.62%; *p* < 0.0001), chronic kidney disease (CKD, i.e., with a glomerular filtration rate <60 mL/min/1.73 m2) (22.66% vs. 11.99%; *p* < 0.0001), Chronic obstructive pulmonary disease (COPD, 10.41% vs. 6.47%; *p* < 0.0001). On the contrary, despite no difference in the prevalence of reduced left ventricular ejection fraction (CIEDI 31.49% vs. 32.99%), history of chronic heart failure was more prevalent in subjects undergoing TLE for non-infective indications (46.95% vs. 42.68%; *p* = 0.011).

Focusing on the 1850/3555 (52.04%) who underwent TLE for CIEDI: 1170/1850 (63.24%) with Lo-CIEDI and 680/1850 (36.76%) with Sy-CIEDI. Table 1 reports baseline characteristics of the 1850 patients according to the type of CIEDI. Patients with Sy-CIEDI were younger (age ≥ 65 years: 66.32% vs. 70.94%, *p* = 0.0380) and with less prevalent obesity (BMI ≥25 Kg/m2 57.16% vs. 62.93%; *p* = 0.0156) and presented more comorbidities such as: reduced left ventricular ejection fraction (LVEF ≤ 35%: 34.57% vs. 29.66%; *p* = 0.0332), history of chronic heart failure (CHF: 45.78% vs. 40.88%; *p* = 0.0406), diabetes mellitus (33.98% vs. 23.92%; *p* < 0.0001) and chronic kidney disease (30.92% vs. 17.87%; *p* < 0.0001). 

### 3.2. CIED History and Characteristics

Unsurprisingly the higher prevalence of chronic heart failure in patients with non-infective indications of TLE was associated with a higher frequency of defibrillators among the implanted devices (53.18% vs. 41.89%; *p* < 0.0001). However, devices for cardiac resynchronization therapy (either defibrillator or pacemaker) were more frequent in patients with CIEDI (25.30% vs. 16.10%; *p* < 0.0001). Patients with non-infective indications underwent TLE after their first implant (57.10% vs. 31.41%; *p* < 0.0001) and with the lesser prevalence of previous CIED-related complications (22.40% vs. 39.62%; *p* < 0.0001) which more frequently implicated a previous malfunction (82.79% of all previous complications), while patients with CIEDI presented a previous infection as the principal cause of previous complication (63.17% of all previous complications).

Compared to Lo-CIEDI, Sy-CIEDI patients presented only minor differences in the implanted hardware at the time of TLE (Table 2), with a slightly higher prevalence of implantable defibrillators (ICD: 44.85% vs. 40.17%; *p* = 0.0490) with similar prevalence of biventricular ICD (21.18% vs. 20.43%; *p* = 0.7014) and the number of leads to be extracted (median 2.00 IQR 2.00–3.00 vs. 2.00 IQR 2.00–3.00; *p* = 0.2993). Notably, patients with Sy-CIEDI more often developed CIEDI after their initial CIED implant compared to patients with Lo-CIEDI (43.24% vs. 24.53%; *p* < 0.0001). However, patients with Lo-CIEDI had a more complex CIED history with a half more previous CIED complications (44.96% vs. 30.44%; *p* < 0.0001) and more revisions of the CIED system (49.15% vs. 28.09%; *p* < 0.0001). Finally, no difference was present in terms of elective box exchange as the last procedure before CIEDI (Lo-CIEDI 17.8% vs. Sy-CIEDI 21.0%; *p* = 0.0855) or prevalence of previous upgrades (Lo-CIEDI 14.96% vs. Sy-CIEDI 12.79%; *p* = 0.1982).

### 3.3. History of Infection and Investigations

Obviously, patients with Sy-CIEDI presented higher WBC counts, average C reactive protein concentration, and prevalence of positive blood cultures and masses (i.e., possible vegetations) at echocardiography which are confirming the presence of systemic involvement of the infectious process (Table 3). Notably, Sy-CIEDI and Lo-CIEDI had an inverse ratio of cultured Staphylococcus species, with a higher prevalence of S. Aureus in Sy-CIED (42.83% vs. 33.23%; *p* = 0.0049). More interestingly, the median time from the first manifestation of infection to enrollment was longer in the Lo-CIEDI group (45 days IQR 17–115 vs. 34 days IQR 15–87; *p* = 0.0027), coupled with a slightly shorter pre-TLE antibiotic treatment (10 days IQR 4–20 vs. 12 IQR 7–23; *p* < 0.0001). Finally, there was also a trend toward a longer time from the last implanted lead to the first sign of CIEDI in Lo-CIEDI patients (47 months IQR 14–96 vs. 43 months IQR 13–87; *p* = 0.0768).

### 3.4. In-Hospital Mortality and Major Complications

**Figure 1:**
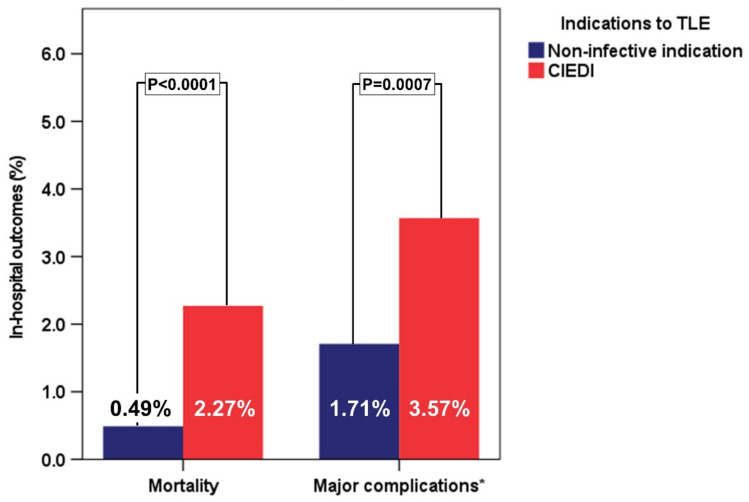
In-hospital clinical outcomes in patients undergoing TLE according to the presence/absence of CIEDI. Legend: CIEDI = CIED infection; TLE = transvenous lead extraction. * = including mortality.

Patients with CIEDI had a worse in-hospital prognosis in terms of major complications (3.57% vs. 1.71%; *p* = 0.0007) and mortality (2.27% vs. 0.49%; *p* < 0.0001) when compared to patients without CIEDI (Figure 1)

**Figure 2:**
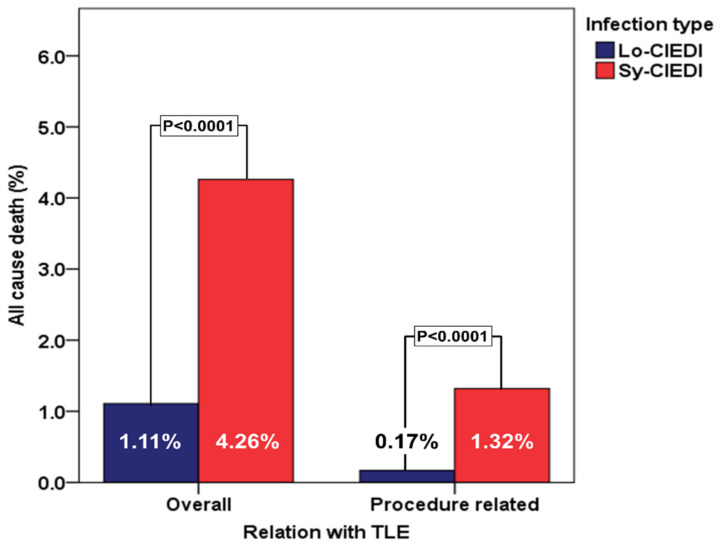
In-hospital procedure-related and non-procedure-related mortality in patients with Sy-CIEDI and Lo-CIEDI (for a detailed description of these cases see [3]). Legend: Lo-CIEDI = local CIED infection; Sy-CIEDI = systemic CIED infection; TLE = transvenous lead extraction.

**Figure 3:**
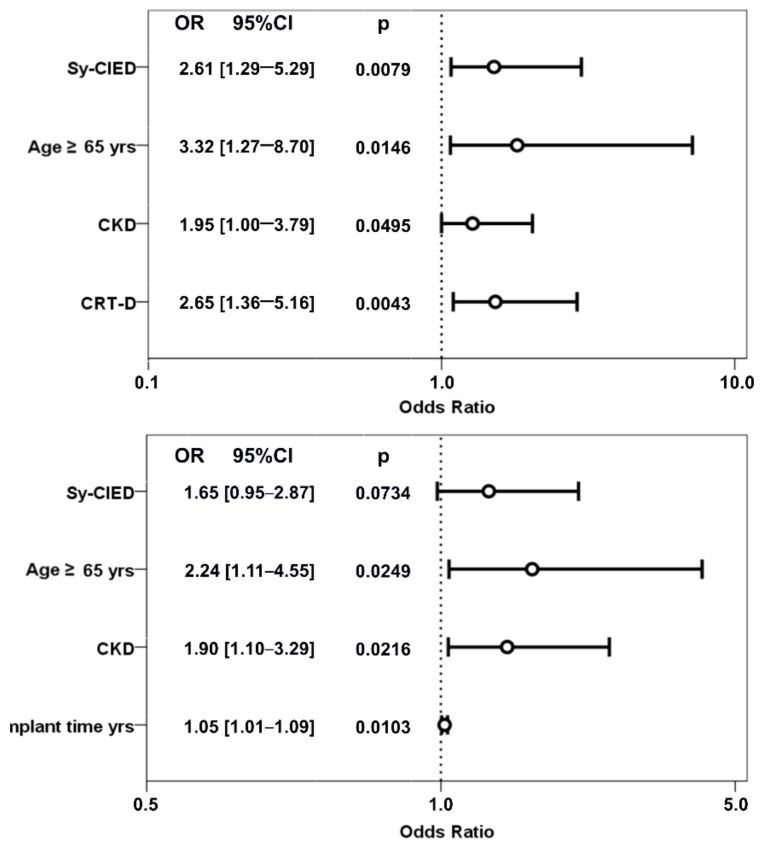
Independent predictors of in-hospital overall mortality and major complications among patients with CIEDI. Legend: CI = confidence intervals; CKD = Chronic Kidney Disease; CRT-D = implantable defibrillator with resynchronization function; HR = hazard ratio; Lo-CIEDI = local CIED infection; Sy-CIEDI = systemic CIED infection; TLE = transvenous lead extraction; yrs = years.

Within the group of patients with CIEDI, in-hospital mortality was significantly higher (*p* < 0.0001) in patients with Sy-CIEDI 29/680 (4.26%) vs. 13/1170 (1.11%) in patients with Lo-CIEDI (Figure 2). Considering all patients with CIEDI, procedure-related mortality was significantly lower than non-procedure related events (11/1850 (0.59%) vs. 31/1850 (1.68%); *p* = 0.0019. Moreover, patients with Sy-CIEDI presented both a significantly higher procedure-related mortality (1.32% vs. 0.17%; *p* < 0.0001) and non procedure-related mortality (4.26% vs. 1.11%; *p* < 0.0001) (Figure 1). The independent predictors of overall in-hospital mortality, in the entire CIEDI population, were the presence of Sy-CIEDI, age ≥65 years, associated chronic kidney disease, and an extraction of a CRT-D device (Table 4, Figure 3).

**Figure 4:**
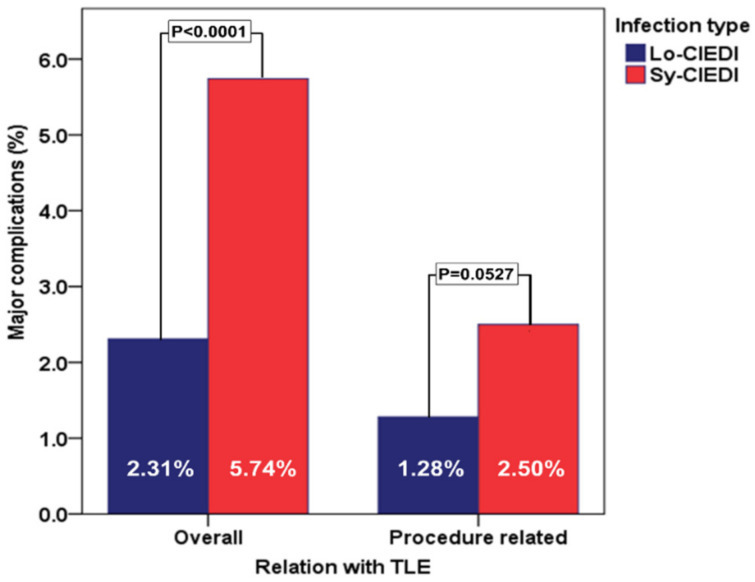
In-hospital major complications in patients with Sy-CIEDI and Lo-CIEDI. Legend: Lo-CIEDI = local CIED infection; Sy-CIEDI = systemic CIED infection; TLE = transvenous lead extraction.

Considering the occurrence of in-hospital major complications (according to pre-specified definition [5]: patients with Sy-CIED had a significantly higher incidence compared with Lo-CIEDI (5.74% vs. 2.31%; *p* < 0.0001), which was just below significance when the analysis was limited to TLE-related events (2.50% vs. 1.28%; *p* = 0.0527) (Figure 4). The independent predictors of in-hospital major complications, in the entire CIEDI population, were age ≥ 65 years, associated chronic kidney disease, and a longer indwelling time (Figure 2). 

## 4. Discussion

The ELECTRa registry was the first large prospective European independent registry on TLE [3]. The principal analysis showed that TLE was an effective procedure (>95% considering both clinical and radiological success) with relatively low procedure-related complications (i.e., 1.7% procedure-related major complications and 0.5% procedure-related mortality). However, complications not related to TLE were relevant, with an overall in-hospital mortality of 1.4% and a 2.7% incidence of in-hospital major complications. Our analysis was aimed at characterizing the subgroup of patients undergoing TLE for CIEDI, which represent about half of the overall candidates for TLE according to current practice [11]. The results highlight the negative impact of Sy-CIEDI on in-hospital mortality and major complications both procedure-related and non-procedure-related. First, the presence of Sy-CIEDI was one of the strongest predictors of overall mortality since 11 out of 17 procedure-related deaths occurred in patients treated for CIEDI, with 9/11 with Sy-CIEDI. Moreover, the multivariate analysis evidenced that a patient age ≥ 65 years and the presence of chronic kidney disease are predictors of major complications and death, independently of the extension of CIEDI. This can be related to the previously described phenomenon of “delayed shock” after TLE, which usually affects patients with Sy-CIEDI and renal failure [12]. All these findings are relevant for patient management following TLE. Until recent years all the focus of the management of CIEDI was the timing and the approach to TLE, however recent literature has shown that, while the timing of TLE is relevant for prognosis, post-procedure mortality is several times higher than procedure-related mortality, being 10–15% at 1 year [9,13]. This information has a significant impact on post-TLE management, including reimplantation strategy [14]. For the same reason, several authors are moving to leadless devices in the hope of providing better outcomes [15,16]. Among the various factors independently associated with long-term mortality chronic kidney disease is one of the more important, as reported by several authors [9,12], both in the acute and long-term settings. Notably, a younger age has usually been reported as a negative predictor for TLE procedures [11], but according to the main results of the ELECTRa registry and the current analysis, this finding was not confirmed [3]. On the contrary, older patients presented a worse prognosis, probably due to comorbidities or maybe secondary to a decreased immune response to CIED infections. The importance of comorbidities is also underlined by the predictive role of CRT-D for in-hospital mortality. This could be the result of a lower left ventricular ejection fraction and a higher prevalence of congestive heart failure. However, both variables had only borderline significance in univariate analysis and were not independent predictors of clinical in-hospital outcomes. A possible criticism of our results can involve the adopted definition of systemic vs. local CIEDI, since as can be seen in the baseline characteristics some patients with Lo-CIEDI presented intracardiac masses or positive blood cultures. However, as discussed in the methods section the choice of local vs. systemic involvement was based on the overall clinical judgement by the study investigator. The motivation belongs on possible confounders with either false positive and false negative deriving by both echocardiography and cultures [4,9,11]. Notably, the decision of the study board was in line with the later Consensus on lead extraction [7], which underlines the role of the experts in lead extraction and CIED infections in the assessment of the presence and type of CIEDI. Notably, neither presence of cardiac masses nor positive blood cultures were predictors of outcomes in univariate and multivariate analyses. Our current analysis would suggest an interesting hypothesis with relevant reflections for the management of patients with CIEDI: Sy-CIEDI seems to be “systemic from the beginning” and less frequently a progression of Lo-CIEDI, at least in a relevant subgroup of patients. This hypothesis derives from the integration of the following data: (1) time from the first sign of infection to enrolment (which usually means referral for TLE) which was significantly higher in Lo-CIEDI (which could theoretically be affected by clinical judgment albeit this is improbable for the high level referral centers involved in the registry with a solid hub-and-spoke organization and awareness of the complications connected with any delay for TLE), (2) the relatively low prevalence of CIED revision and complications coupled with a higher prevalence of first implants in Sy-CIEDI patients (with similar prevalence of upgrades), (3) the increased prevalence of several comorbidities known to be associated with septic shock and endocarditis in Sy-CIEDI (i.e., diabetes, chronic kidney disease and CHF) [17,18]. On the opposite progression from a local infection to a Sy-CIEDI is expected to occur after several procedures involving CIED pocket and longer (but ineffective) attempts of antibiotic treatments and local revisions. Our results are in keeping with the findings of Greenspon et al. who analyzed the differences between patients presenting with lead-related endocarditis with early vs. late development from the last CIED procedure adopting a cut-off of six months [19]. In their cohort, patients with delayed manifestations of CIEDI (>70%) had a prevalence of S.Aureus (41.2%) and Coagulase-negative Staphylococci (24.5%) in keeping with our results. Moreover, patients with delayed lead-related endocarditis frequently developed CIEDI after the first implant (54.9% vs. 34.9%; *p* = 0.03). Certainly, our results can be currently considered only as hypothesis generating but if the suspicion that in a relevant subgroup of patients Sy-CIEDI and Lo-CIEDI are not two steps of the same process while they are two different diseases this will heavily impact on CIEDI management, from prophylaxis until post-TLE management [2,9]. This concept is further supported by a recently published single centre prospective study involving 105 patients undergoing an 18F-FDG PET/CT scan before TLE to improve post-TLE patient management and assess the role of this exam in improving the prediction of post-TLE survival [20]. In this cohort, 24/105 patients presented a particular pattern called “Cold Closed Pocket” defined as the presence of CIED-related infection without CIED pocket involvement (i.e., without skin erosion/perforation nor increased capitation at 18F-FDG PET/CT scan) which was the only predictor of long term survival together with renal failure. Notably, patients with a Cold Closed Pocket less frequently had generator replacement (37.5% vs. 64.2%; *p* = 0.020) while they were more frequently at their first implant (54.2% vs. 24.7%; *p* = 0.006), without difference in terms of implanted hardware. Moreover, they presented more frequently ghosts after TLE (33.3% vs. 8.6%; *p* = 0.002) despite a trend of a lower lead indwelling time and no difference in terms of anticoagulant use. All these findings suggest that in a range between one-fifth to one quarter of CIEDI patients another possible mechanism can be involved, maybe as previously suggested Sy-CIEDI could develop independently from Lo-CIEDI through direct seeding during intermittent bacteremia upon leads with higher abrasion [10,21]. In this light, it is important for the interpretations of the results of the World-wide Randomized Antibiotic EnveloPe Infection PrevenTion (WRAP-IT) randomized trial [22], evaluating the efficacy of the TYRX™ Absorbable Antibacterial Envelope to prevent CIEDI as previously evidenced in several pivotal experiences [23]. Despite the selection of candidates with a lower chance of systemic CIEDI the subgroup of patients undergoing their first CRT-D implant experienced more (albeit not significant) infections in the envelope group (7/536 vs. 3/586), while patients undergoing replacement/upgrade/revision presented the greater benefit (11/2098 vs 32/2033) [24]. These considerations deserve additional data to tailor CIEDI prophylaxis according to patient and procedural characteristics [24,25].

## 5. Limitations

The ELECTRa findings are subject to the limitations inherent to observational studies, including the possibility of unknown confounders and bias in management strategy. To ensure data integrity source data and database quality control was performed by dedicated data monitors to ensure that all consecutive patients were included in participating centres. The participation in the ELECTRa registry was based on a voluntary basis: and complication rates may therefore be underestimated since there are centres, physicians, and surgeons performing lead extraction that did not participate in the Registry. Although there was participation from all of the major centres/countries performing extraction the patients recruited may not represent the daily practice of lead extraction in all countries where a high proportion of lead extraction is undertaken by non-EHRA affiliated centres. Similarly, patients with an indication for TLE who were referred for open surgical extraction were excluded from the study. The purpose of ELECTRa was to offer a multicentre prospective overview of TLE safety and efficacy in Europe. Albeit it was concluded in 2014 the approach to lead extraction still reflects current practice. Predictors of outcomes were identified and discussed although the exact cause-effect relationships remain speculative. At the same time, ELECTRa was not a registry focusing on CIEDI but on TLE with the possible bias of leaving unexplored patients who were not candidates to TLE. For this reason, it did not consider diagnostic procedures like PET/CT scans [26]. However, TLE is the current standard of care of CIEDI and ELECTRa provide prospective data on currently treated candidates to TLE, which underlines the relevance of our results. Finally, as previously acknowledged the distinction between Sy-CIEDI and Lo-CIEDI in a specific patient was mainly based on the interpretation of the complete clinical picture by each investigator, leading to the possibility of misclassification. However, this approach reflects the current clinical practice and the limitation in univocally defining local vs. systemic CIEDI [8,26,27].

## 6. Conclusions

CIEDI is the most frequent indication for TLE in current clinical practice in Europe and it is associated with worse in-hospital outcomes. In particular patients with Sy-CIEDI have an increased risk of procedure-related and overall in-hospital mortality. Patients with Sy-CIEDI have more multiple comorbidities. Several clinical and instrumental factors suggest that in a relevant subgroup of patients, Sy-CIEDI at the time of TLE was not a progression from Lo-CIEDI. This hypothesis if confirmed by ongoing studies will have relevant impact on the pre- and post-procedure management of candidates for TLE and CIEDI prophylaxis.

## Figures and Tables

**Table 1:** Baseline clinical characteristics of patients with Lo-CIEDI vs. Sy-CIEDI.

Patient characteristics	Lo-CIEDI (1170 pts.)	Sy-CIEDI (680 pts.)	*p*
General characteristics, N/Total N (%)			
Male sex	922/1170 (78.80%)	520/680 (76.47%)	0.2432
**Age ≥ 65 years**	**830/1170 (70.94%)**	**451/680 (66.32%)**	**0.0380**
**BMI > 25 Kg/m^2^**	**725/1152 (62.93%)**	**375/656 (57.16%)**	**0.0156**
Hi-vol centre	964/1170 (82.39%)	545/680 (80.15%)	0.2296
**LVEF ≤ 35%**	**323/1089 (29.66%)**	**223/645 (34.57%)**	**0.0332**
**Anticoagulation pre-TLE**	**428/1170 (36.58%)**	**309/680 (45.44%)**	**0.0001**
Heart disease N/Total N (%)			
Coronary artery disease	451/1160 (38.88%)	282/670 (42.09%)	0.1769
Valvular heart disease	179/1167 (15.34%)	124/678 (18.29%)	0.0990
Dilated cardiomyopathy	313/1165 (26.87%)	184/673 (27.34%)	0.8258
Hypertrophic cardiomyopathy	40/1168 (3.42%)	32/678 (4.72%)	0.1658
Primary electrical disease	362/1164 (31.10%)	182/675 (26.96%)	0.0610
**CHF**	**475/1162 (40.88%)**	**309/675 (45.78%)**	**0.0406**
Comorbidities N/Total N (%)			
Hypertension	697/1162 (59.98%)	392/673 (58.25%)	0.4655
**Diabetes**	**278/1162 (23.92%)**	**229/674 (33.98%)**	<**0.0001**
COPD	115/1162 (9.90%)	76/673 (11.29%)	0.3453
**Chronic kidney disease**	**208/1164 (17.87%)**	**209/676 (30.92%)**	<**0.0001**

Legend: BMI = body mass index; CHF = chronic heart failure; CIED = cardiac implantable electrical device; COPD= Chronic obstructive pulmonary disease; Hi-vol = High volume(≥30 TLE procedures/year); Lo-CIEDI = local CIED infection; LVEF = left ventricular ejection fraction; N = number; pts.= patients; Sy-CIEDI = systemic CIED infection; TLE= transvenous lead extraction. In **bold** are reported characteristics with a *p* value < 0.05.

**Table 2:** CIED history and characteristics in patients with Lo-CIEDI vs. Sy-CIEDI.

CIED Characteristics	Lo-CIEDI (1170 pts.)	Sy-CIEDI (680 pts.)	*p*
General characteristics, N/Total N (%)			
**ICD**	**470/1170 (40.17%)**	**305/680 (44.85%)**	**0.0490**
CRT-D	239/1170 (20.43%)	144/680 (21.18%)	0.7014
PM dependency	318/1170 (27.18%)	157/680 (23.09%)	0.0521
Patients with >2 target leads	393/1170 (33.59%)	212/680 (31.18%)	0.2860
CIED history, N/Total N (%)			
**Previous CIED complication: overall**	**526/1170 (44.96%)**	**207/680 (30.44%)**	**<0.0001**
Previous CIED complication: infective	337/526 (64.07%)	126/207 (60.87%)	0.4189
Previous CIED complication: malfunction	192/526 (36.50%)	85/207 (41.06%)	0.2516
Previous CIED complication: thrombotic	9/526 (1.71%)	6/207 (2.90%)	0.3066
**Last procedure: CIED implant**	**287/1170 (24.53%)**	**294/680 (43.24%)**	**<0.0001**
Last procedure: CIED replacement	208/1170 (17.77%)	143/680 (21.03%)	0.0855
**Previous CIED revision**	**575/1170 (49.15%)**	**191/680 (28.09%)**	**<0.0001**
Previous CIED upgrade	175/1170 (14.96%)	87/680 (12.79%)	0.1982

Legend: CIED = cardiac implantable electrical device; CRT-D = implantable defibrillator with resynchronization therapy; ICD = implantable cardioverter defibrillator; Lo-CIEDI = local CIED infection; N = number; PM = pacemaker; pts.= patients; Sy-CIEDI = systemic CIED infection; TLE = transvenous lead extraction. In **bold** are reported characteristics with a *p* value <0.05.

**Table 3:** Clinical and instrumental data on CIED infection in patients with Lo-CIEDI vs. Sy-CIEDI.

Infection Chacracteristics	Lo-CIEDI (1170 pts.)	Sy-CIEDI (680 pts.)	*p*
Instrumental examination, N/Total N (%) or Median [IQR]			
Masses at TTE/TEE *	**121/1170 (10.34%)**	**445/680 (65.44%)**	**<0.0001**
**WBC count (x10e9/L)**	**7.20 (5.99–8.63) (N = 1095)**	**8.08 (6.30–10.60) (N = 629)**	**<0.0001**
**C-reactive protein (mg/L)**	**4.00 (1.40–12.00) (N = 977)**	**17.00 (4.50–65.00) (N = 581)**	**<0.0001**
Blood cultures, N/Total N (%)			
Positive culture **	**328/958 (34.24%)**	**544/659 (82.55%)**	**<0.0001**
**Coagulase-negative Staphylococcus**	**140/328 (42.68%)**	**185/544 (34.01%)**	**0.0102**
**Staphylococcus Aureus**	**109/328 (33.23%)**	**233/544 (42.83%)**	**0.0049**
Other agents	99/328 (30.18%)	161/544 (29.60%)	0.8542
Infection history and antibiotic treatment, N/Total N (%) or Median [IQR]			
**Time 1st sign of infection** **—enrolment (days)**	**45.00 (17.00–115.00)** **(N = 1113)**	**34.00 (15.00–87.00)** **(N = 663)**	**0.0027**
Time from last lead implanted to 1st sign of infection (months)	47 (14–96) (N = 1094)	43 (13–87) (N = 649)	0.0768
**Antibiotic pre-TLE**	**854/1170 (72.99%)**	**636/680 (93.53%)**	**<0.0001**
**Empirical antibiotic treatment**	**645/854 (75.53%)**	**186/636 (29.25%)**	**<0.0001**
**Blood Culture-guided antibiotic treatment**	**81/854 (9.48%)**	**430/636 (67.61%)**	**<0.0001**
**Duration of pre-TLE antibiotic**	**10.00 (4.00–20.00)** **(N = 791)**	**12.00 (7.00–23.00)** **(N = 596)**	**<0.0001**

* the difference from thrombus/vegetation was made on investigator discretion and classification (Lo-CIEDI vs. Sy-CIEDI) was performed by the investigator in accordance. ** at least one positive culture. Legend: CIED = cardiac implantable electrical device; IQR = interquartile range; Lo-CIEDI = local CIED infection; N = number; pts.= patients; Sy-CIEDI = systemic CIED infection; TEE = trans-oesophageal echocardiography; TLE= transvenous lead extraction; TTE = trans-thoracic echocardiography; WBC = white blood cells. In **bold** are reported characteristics with a *p* value < 0.05.

**Table 4:** Predictors of in-hospital mortality and major complications at univariate cox-regression analysis in a patient undergoing TLE for CIEDI (1850/3555 patients).

Covariable	Mortality	Major Complications
	**Hazard-Ratio [95% CI]**	
Age ≥ 65 years	2.64 (1.17–6.00)	2.23 (1.21–4.12)
LVEF ≤ 35%	2.06 (1.12–3.81)	n.s.
Chronic Heart Failure	1.98 (1.05–3.73)	n.s.
Chronic kidney disease	2.94 (1.60–5.42)	2.23 (1.36–3.65)
PM dependency	n.s.	2.06 (1.26–3.36)
CRT-D	2.45 (1.32–4.53)	n.s.
Oldest lead dwelling time (years)	n.s.	1.06 (1.02–1.10)
Number of leads ≥ 3	2.21 (1.19–4.10)	n.s.
Sy-CIEDI	2.82 (1.45–5.48)	1.79 (1.08–2.97)

Legend: CI = confidence interval; CRT-D = cardiac resynchronization defibrillator; LVEF= left ventricular ejection fraction; Lo-CIEDI = local CIED infection; PM = pacemaker; Sy-CIEDI = systemic CIED infection; TLE= transvenous lead extraction.

## Data Availability

Restrictions apply to the availability of these data. Eventual permission to access the original data have to be presented to the European Heart Rhythm Association (EHRA).

## Supplementary Materials

The following are available online at https://www.mdpi.com/article/10.3390/biology11040615/s1, File S1: ELECTRa registry Principal Investigators by Countries.
