# Supplementary material for: Transvenous Lead Extraction in Patients with Cardiac Implantable Device: The Impact of Systemic and Local Infection on Clinical Outcomes—An ESC-EHRA ELECTRa (European Lead Extraction Controlled) Registry Substudy
Source: Biology (Basel). 2022 Apr 18;11(4):615. doi: 10.3390/biology11040615 (PMC9033150; doi:10.3390/biology11040615)
Supplement: Supplementary file 1 [file biology-11-00615-s001.zip › biology-1558312-supplementary.pdf]

ELECTRa registry Principal Investigators by Countries:

AZERBAIJAN *Baku :* Dr. F. Aliyev ;

BELGIUM *Aalst* **:** Dr. B. Stockman ; Dr. F. Casselman ; Dr. R. Beelen; *Brussels* **:** Dr. J. Czapla ;

Ghent **:** Dr. F. Van Heuverswyn ; *Liege* **:** Dr. Q. Desiron ;

DENMARK *Aalborg* **:** Prof. O. Eschen ; Dr. Thogersen; *Aarhus* **:** Dr. P. T. Mortensen ; *Copenhagen :* Dr. J. Svendsen ; Dr. H. Høgh Petersen ; Dr. B. Thornvig Philbert ;

FRANCE *Brest :* Prof. J. Mansourati ; Dr M. Fatemi ; *Dijon :* Prof. F. Garnier ; Dr C. Guenancia ; Dr G. Laurent ; *Grenoble :* Dr. P. Defaye ; Dr. P. Jacon; *Lille :* Dr. C. Marquie ;Prof. D. Klug ; Dr. Z. Soussi ; Dr. D. Lacroix ; *Marseille :* Prof. F. Franceschi ; Dr. S. Prévôt ; *Massy :* Prof. J. Horvilleur ; .Dr. F. Salerno, Dr. V. Manenti, Dr. J. Lacotte ; *Montpellier :* Prof. J-L. Pasquie ; Dr F. Massin ; Dr T. Tri Cung ; *Paris :* Dr. F. Hidden-Lucet ; Dr D'Alessandro Cosimo ; Dr E. Gandjbakhch ; Dr G. Duthoit ; *Rennes :* Prof. C. Leclercq ; Dr. N. Behar ; Dr. P. Mabo ; *Rouen :* Prof. F. Anselme; Dr. K Garsey; Dr. B. Godin Gardea; Dr. A. Savoure ; *Toulouse :* Dr. B. Casteigt ;

GERMANY *Bad Nauheim :* Dr. H. Burger ;Prof. T. Walther ;Prof. T. Ziegelhoeffer ; *Berlin :* Dr. B. Peters ; *Bernau :* Mr. F. Hoelschermann; Dr. Moeller; *Dusseldorf :* Prof. B. Osswald ; *Essen :* Dr. D. Schmitz ; Dr. R. Palea ; Dr. H.

Gueldner ; *Leipzig :* Prof. G. Hindricks ; Dr. S. Richter ; Dr. M. Döring ; *Volkingen :* Prof. K-D. Heib ;

GREAT BRITAIN *London :* Prof. A. Rinaldi ;

GREECE *Athens :* Dr. S. Sideris ; Dr. J. E. Kallikazaros ; Prof. D. Tousoulis ; Prof. K. A. Gatzoulis ; *Platani-Patra :* Prof. J-I. Chiladakis ; *Thessaloniki :* Prof. V.P. Vassilikos ; Prof. N. Frangakis, Dr. E. Pagourelias ;

HUNGARY *Szeged :* Prof. T. Forster ;

ISRAEL *Ramat Gan :* Prof. M. Glikson ; Dr. E. Nof ; Dr. R. Beinart ;

ITALY *Bari :* Dr. G. Luzzi ; Dr. R. Trotta ; Dr. Z. Palama ; *Bergamo :* Dr. G. Mascioli ; Dr. E. Lucca ; Dr. N. Bakhtadze ; Dr. F. Michelotti ; *Bologne :* Dr. I. Diemberger; *Brescia :* Dr. A. Curnis ; *Catania :* Prof. V. Calvi ; Dr. A. Di Grazia ; Dr. A. Arestia ; *Firenze :* Dr. E. V. Dovellini ; Dr. L. Giurlani ; *Genoa:* Dr. G. Bertero ; Prof. C. Brunelli; Dr. A. Porcile; Dr. P. Sartori ; *Lecce :* Dr. E. C.L. Pisano ; Dr. F. Magliari ; Dr. G. Milanese ; Mr. M. Lauretti ; *Venice :* Dr. A. Rossillo ; Dr. G. Gasparini ;Dr. C. Ardito, Dr. P. China ; *Milan:* Dr. G. Magenta ; Dr. C. Ardito ; Dr. S. Vargiu ; Dr. E. Mulargia ; Milan: Dr. P. Della Bella ; *Naples :* Dr. V. Caprioli ; *Novara :* Dr. A. Magnani ; Dr. E. Occhetta ; Prof. P. N. Marino ; *Pavia :* Dr. M. Landolina ; Dr. B. Petracci; Dr. R. Rordorf; Dr. A. Vicentini ; *Pisa :* Dr. A. Di Cori ; Dr L. Segreti ; Dr E. Soldati ; Dr. G. Zucchelli ; Dr. S. Viani ; Dr. L. Paperini ; *Rome :* Prof. A. Ciccaglioni ; Rome : Dr. G. Pelargonio ; Dr. F. Perna ; Dr. G. Bencardino ; Dr. M. L. Narducci ; *Turin:* Dr. P. G. Golzio ; Dr. E. Pellissero ; Dr. F. Gaita ; Dr. D. Castagno ; *Trento :* Dr. L. Gramegna ;

LITHUANIA *Vilnius :* Prof. G. Marinskis ;

NORWAY *Oslo :* Dr. E. S. Platou ; Dr. T. M. Knutsen ; Dr. T. Steen ; Dr. P. Vanberg ;

POLAND *Gdansk :* Dr. M. Kempa ; Dr. S. Budreijko ; *Krakow :* Prof. B. Malecka ; Dr. K. Boczar ; Dr. A. Ząbek ; *Lublin :* Dr. A. Tomaszewski ; Prof. A. Polewczyk ; Dr. M. Czajkowski ; Dr. W. Brzozowski ; Dr. A. Polewczyk Tuchalska ; Dr. M. Polewczyk ; *Poznan :* Prof. P. Mitkowski ; Dr. A. Katarzynska-Szymanska ; Dr. L. Chmielewska-Michalak ; *Warsaw :* Prof. A. Maciag ; Prof. M. Grabowski ; Dr. G. Opolski ; Dr. A. Kołodzińska ; *Wroclaw :* Dr. D. Jagielski ; Dr. K. Nowak ;

PORTUGAL *Lisboa :* Dr. B. Valente ; Prof. M. Martins Oliveira ; Dr. P. Cunha ; Dr. A. Lousinha ;

RUSSIAN FEDERATION *Rostov-on-Don :* Prof. V. Korshunov ;

SPAIN *Barcelona :* Dr. A. Moya ; Dr. I. Roca ; Dr. M. Moradi ; *Madrid :* Prof. I. Fernandez Lozano ;Prof. J. Toquero Ramos ; Prof. V. Castro Urda ; Dr. L. Diego Solis ; *Madrid :* Dr. E. Gonzalez ; Dr. J. Silvestre ; Dr. O. Al Razzo ; *Valencia :* Dr. O. Cano ; Dr. J. Olagüe de Ros ; Dr. M-J. Sancho-Tello de Carranza ; Dr. J. Osca Asensi ;

SWEDEN *Goteborg :* Dr. J. Gäbel ; *Stockholm :* Prof. F. Gadler ; *Uppsala :* Dr. D. Mortsell ; Dr. P. Teder ; Mrs Anette Lorentzon;

SWITZERLAND *Bern :* Prof. J. Fuhrer ; *Lugano :* Dr. F. Regoli ; Dr. M.L. Caputo ; Dr. T. Moccetti ; *Zurich :* Prof. C. Starck ;

UKRAINE *Kiev :* Dr. V. Zalevskiy ;
